# Silicon addition improves plant productivity and soil nutrient availability without changing the grass:legume ratio response to N fertilization

**DOI:** 10.1038/s41598-020-67333-7

**Published:** 2020-06-24

**Authors:** Danghui Xu, Tianpeng Gao, Xiangwen Fang, Haiyan Bu, Qiuxia Li, Xiaona Wang, Renyi Zhang

**Affiliations:** 1 State Key Laboratory of Grassland Agro-Ecosystems/School of Life Science, Lanzhou University, No. 222, South Tianshui Road, Lanzhou, 730000 Gansu China; 2 College of Biological and Environmental Engineering, Xi’an University, Xi’an, 710065 China; 3 The Engineering Research Center of Mining Pollution Treatment and Ecological Restoration of Gansu Province, Lanzhou City University, Lanzhou, 730070 China

**Keywords:** Grassland ecology, Conservation biology

## Abstract

Silicon (Si) plays an important role in plant nutrient capture and absorption, and also promotes plant mechanical strength and light interception in alpine meadows. In this study, we conducted a field experiment to examine the effect of nitrogen (N) application, with (N + Si) and without Si (N-only), on the potential for soil nutrient and the growth of grass and legume plant functional types (PFTs) in an alpine meadow. It was found that N + Si resulted in higher soil nutrient contents, leaf N and P concentrations, abundance and biomass of legume and grass PFTs than N-only. The aboveground biomass of grass (598 g m^−2^) and legume (12.68 g m^−2^) PFTs under 600 kg ha^−1^ ammonium nitrate (NH_4_NO_3_) per year addition with Si was significantly higher than that under the same level of N addition without Si (515 and 8.68 g m^−2^, respectively). The grass:legume biomass ratio did not differ significantly between the N + Si and N-only. This demonstrates that Si enhances N fertilization with apparently little effect on grass:legume ratio and increases plant-available nutrients, indicating that Si is essential for the plant community in alpine meadows.

## Introduction

Nitrogen (N) is often the primary limiting nutrient for plant growth in terrestrial ecosystems, especially in alpine meadows, where the low temperatures and short growing season limit plant growth and nutrient cycling, and thus N fertilization is widely used in these environments^1–3^. Nitrogen fertilization has been reported to influence ecosystems in a variety of ways, including changes in species diversity, biomass production, nutrient availability and soil conditions^1,4–7^.

Silicon (Si) is important for nutrition and nutrient cycling in soil and plants, especially for grassland environments^8–10^. In natural ecosystems, Si fertilization can increase plant growth, plant N use efficiency^7,11–13^ and alleviate the loss of biodiversity induced by N addition^6,14^. The availability of Si during plant growth not only modifies the concentration of nutrient ions such as N and phosphorus (P) in soils^15–17^, but also strongly affects growth and abundance of grass and legume species in grasslands^16,18–20^. Hence, Si could play an important role in plant community composition such as plant abundance and biomass ratios of different plant functional types (PFTs), but has received little research attention^21–23^.

Legume species are an important PFT in grassland. Legumes not only increase soil N and plant biomass^7,12^, but also maintain a balance of dominance of grass and other non-legume species in plant community. Therefore, management involving mixing legumes with grasses provides economic and environmental benefits and is considered a sustainable intensification in grassland^17,19,24^. However, to our knowledge, no studies have examined the impacts of N + Si on soil nutrient ions and growth of grass and legume PFTs (plant abundance, aboveground biomass, leaf N and P concentration) and abundance and biomass grass:legume ratios. In this study, we tested the hypothesis that there are high differences in growth of grass and legume PFTs and soil N and P concentration in an alpine meadow under different levels of N addition without Si (N-only), Si addition only and N added with Si (N + Si), and N + Si has a more beneficial effect than N-only. To test this hypothesis, our objectives were to (1) evaluate the effect of Si or N addition only and the interactive effect of N + Si addition simultaneously on the concentrations of soil ammonium (NH_4_^+^-N), nitrate (NO_3_^−^-N) and available P, (2) identify whether there were any different responses of the growth of grass and legume PFTs under the different fertilizers and (3) determine whether or not there is a beneficial interaction effect of N and Si addition on grass:legume abundance and biomass ratios.

## Results

### Soil nutrient ions and pH

The fertilization of N-only and N + Si did not affect the soil organic carbon (C) and total N concentrations. Addition of 600 kg ha^−1^ ammonium nitrate (NH_4_NO_3_) per year significantly reduced soil pH by 0.35, but Si addition did not affect soil pH (Table S1). There were no significant interaction effects between Si and N on soil pH (two-way ANOVA).

**Figure 1:**
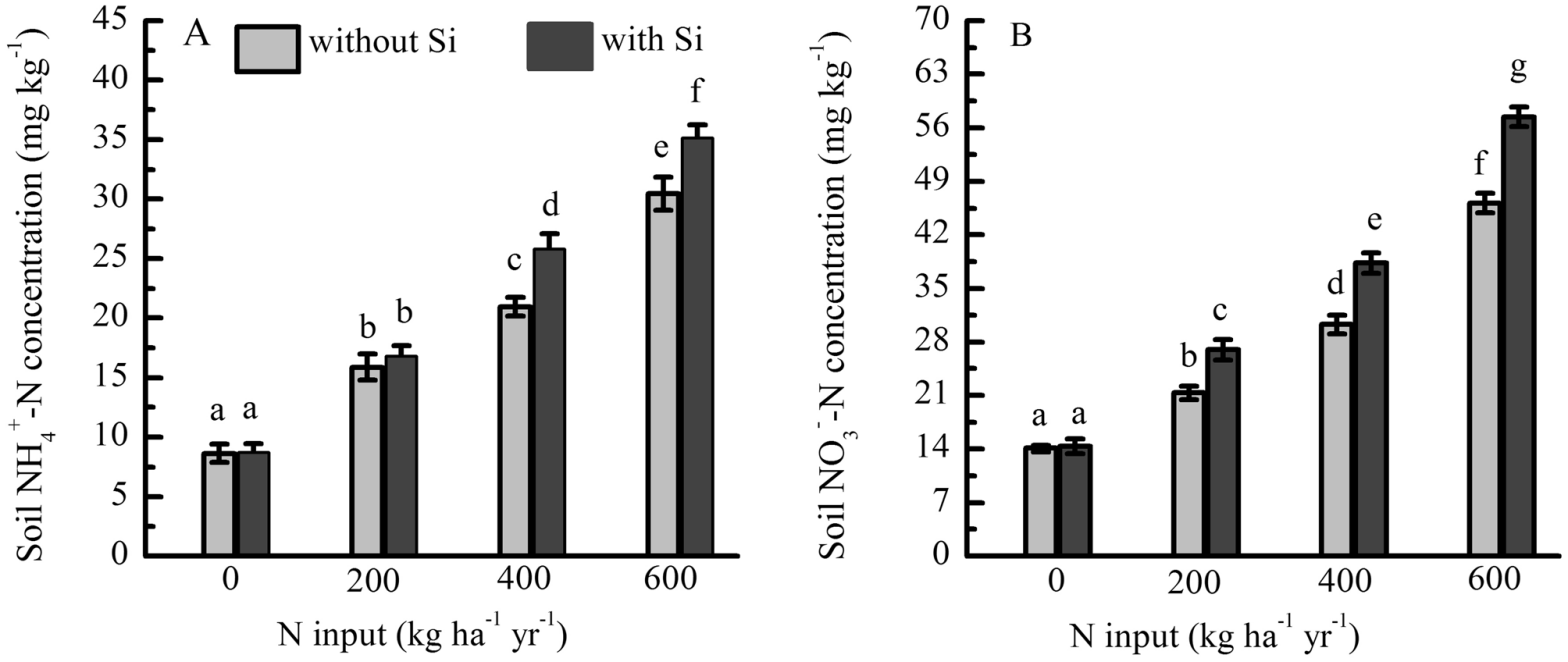
(**A**) Soil NH_4_^+^-N and (**B**) NO_3_^−^-N concentrations under fertilization by N with Si (N + Si) and without Si (N-only) expressed as 3-year averages (2011–2013, *N* = 6).

The fertilization of N-only and N + Si increased soil NH_4_^+^-N and NO_3_^−^-N concentrations (Fig. 1). The soil NH_4_^+^-N and NO_3_^−^-N concentrations increased by 84% and 52%, 142% and 115%, and 252% and 228% relative to the control (unfertilized) plot with the addition of 200, 400 and 600 kg ha^−1^ NH_4_NO_3_ only, respectively. The soil NH_4_^+^-N and NO_3_^−^-N concentrations increased by 93% and 88%, 196% and 167%, and 303% and 299% relative to the control plot with the addition of 200, 400 and 600 kg ha^−1^ NH_4_NO_3_ plus Si, respectively. The interaction between Si and N had significant effects on the soil NH_4_^+^-N (*F* = 36.52, *P* < 0.001) and NO_3_^−^-N (*F* = 110, *P* < 0.001) concentrations.

**Figure 2:**
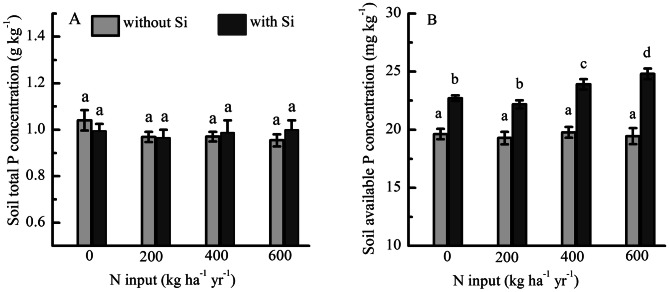
(**A**) Soil total P and (**B**) available P concentrations under fertilization by N with Si (N + Si) and without Si (N-only) expressed as 3-year averages (2011–2013, *N* = 6).

Experimental additions of N-only had no effect on soil total P concentration (*F* = 1.938, *P* = 0.156) (Fig. 2A) and soil available P (*F* = 0.159, *P* = 0.923) (Fig. 2B) relative to the control plot. The soil available P concentration increased significantly with increasing inputs of N + Si, with an increase of 15.70%, 20.83% and 27.51%, respectively, with addition of N + Si relative to the addition of N-only (Fig. 2B). There was a highly significant interaction between Si and N (*F* = 3.171, *P* = 0.034) in the soil available P concentration.

### Aboveground biomass, abundance of the grass and legume PFTs

**Figure 3:**
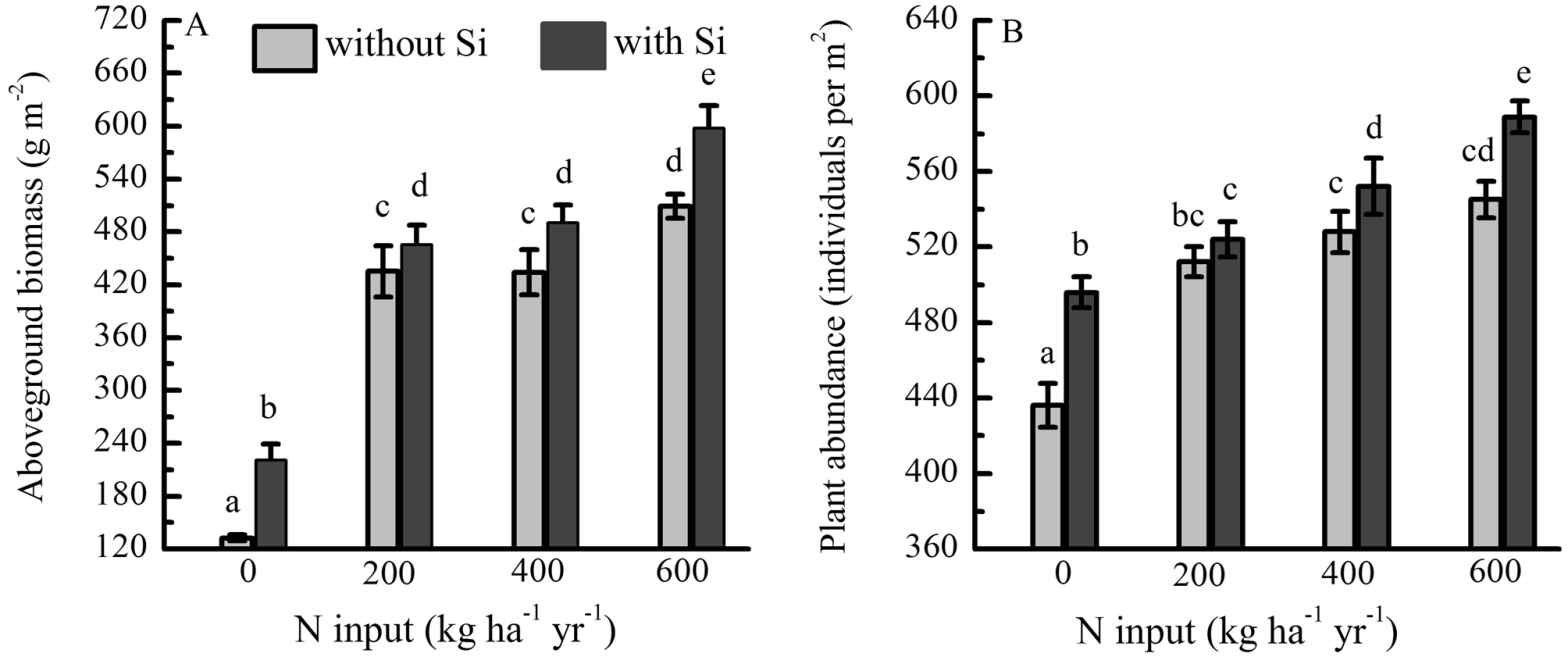
(**A**) Aboveground biomass and (**B**) abundance of grass PFTs under fertilization by N with Si (N + Si) and without Si (N-only) expressed as 3-year averages (2011–2013, *N* = 6).

The aboveground biomass (from 133 to 509 g per m^2^) (Fig. 3A) and plant abundance (from 436 to 545 individuals per m^2^) (Fig. 3B) of the grass PFTs increased with increasing rates of N. The addition of N + Si led to a significantly higher aboveground biomass and abundance than addition N-only (Fig. 3). The aboveground biomass (589 g per m^2^) under N addition with 600 kg ha^−1^ NH_4_NO_3_ per year plus Si was significantly higher than that (509 g per m^2^) under the same level of N addition without Si. There were significant interaction effects between Si and N in the aboveground biomass and abundance (two-way ANOVA analysis).

**Figure 4:**
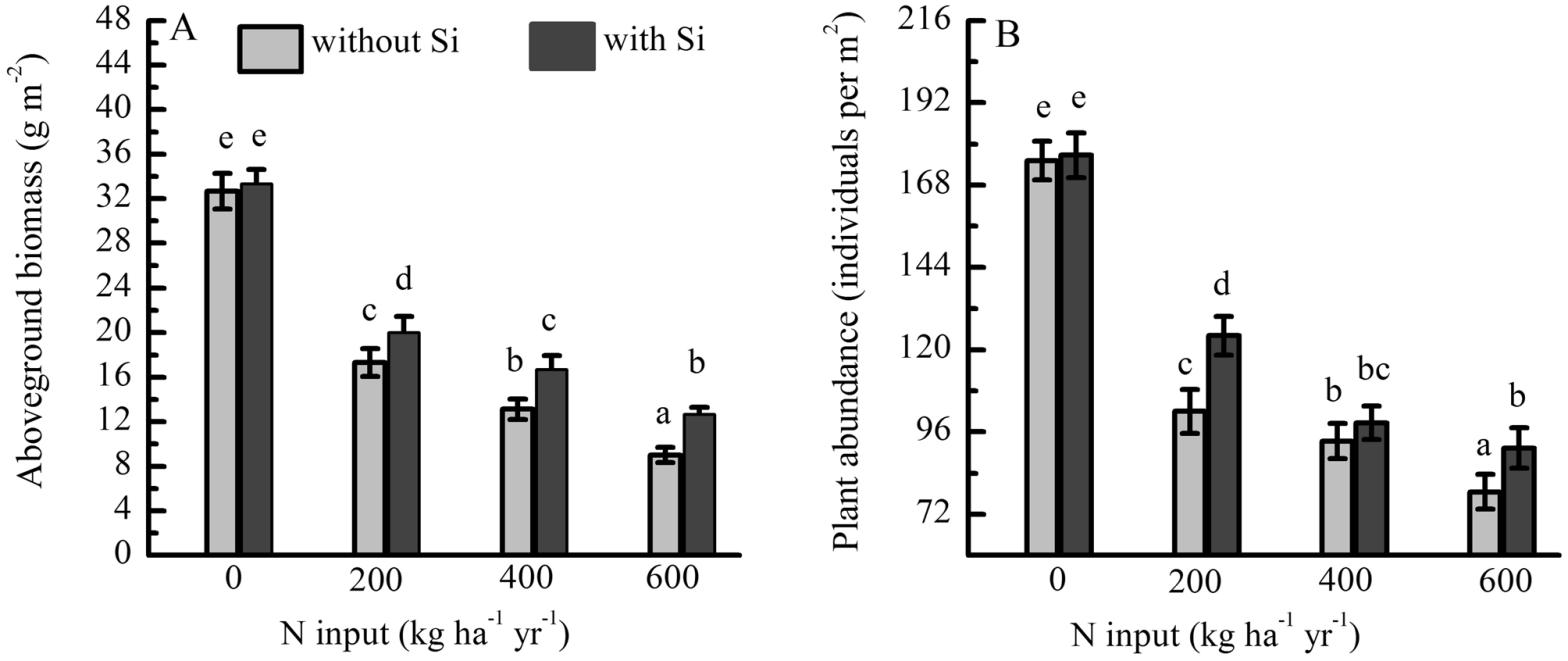
(**A**) Aboveground biomass and (**B**) abundance of legume PFTs under fertilization by N with Si (N + Si) and without Si (N-only) expressed as 3-year averages (2011–2013, *N* = 6).

The aboveground biomass (from 32.68 to 8.64 g per m^2^) (Fig. 4A) and abundance (from 350 to 78.52 per m^2^) (Fig. 4B) of the legume PFTs decreased with increasing rates of N application from control to N addition with 600 kg ha^−1^ NH_4_NO_3_. The aboveground biomass (12.68 g per m^2^) under N addition (600 kg ha^−1^ NH_4_NO_3_) with Si was significantly higher than that (8.68 g per m^2^) under the same level of N addition without Si. The interaction of N fertilization with Si had significant effects on plant abundance of the legume PFTs.

### Leaf N and P concentration of grass and legume PFTs

**Figure 5:**
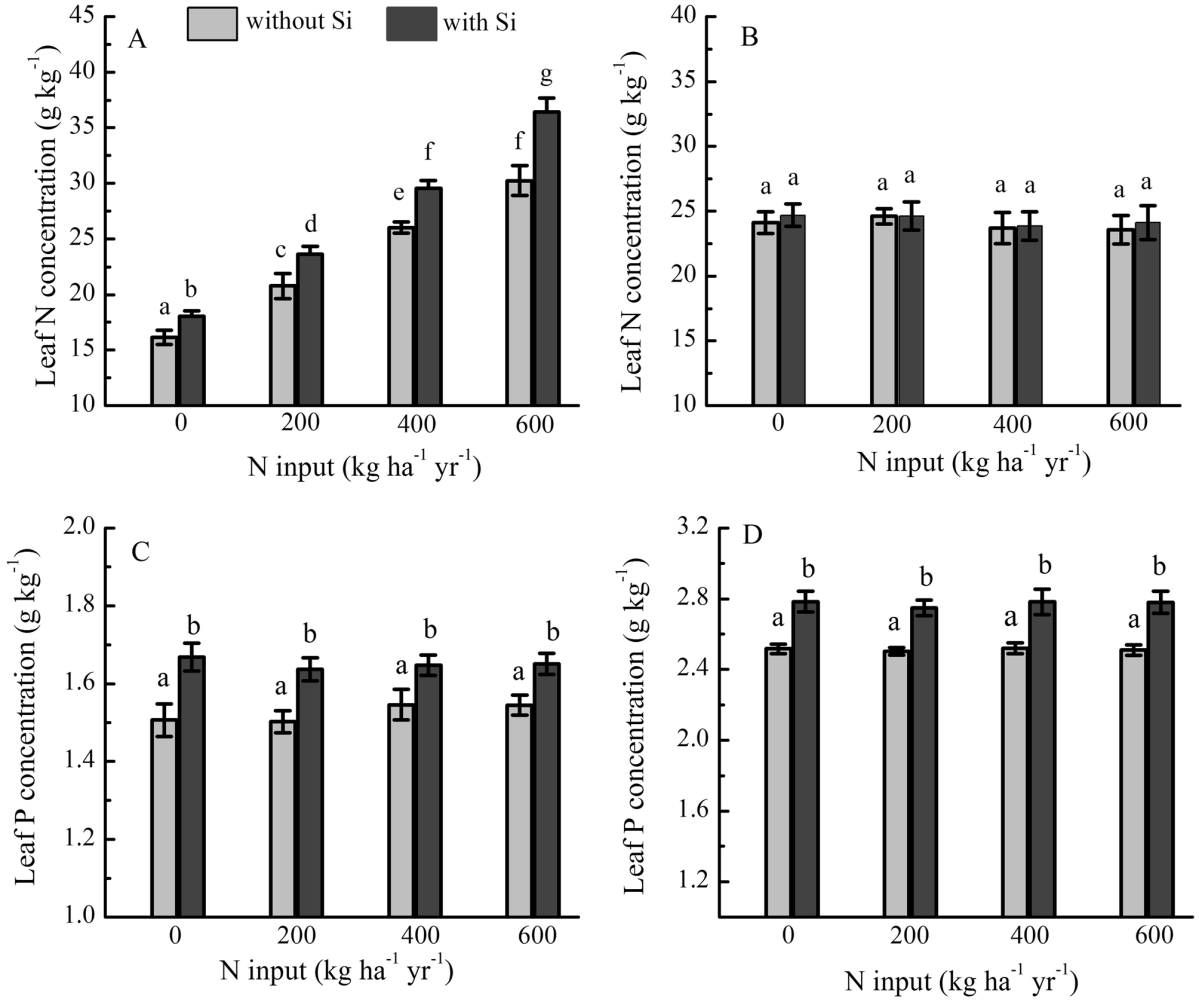
(**A**, **B**) Leaf N concentration and (**C**, **D**) leaf P concentration of grass (left) and legume (right) PFTs under fertilization by N with Si (N + Si) and without Si (N-only) expressed as 3-year averages (2011–2013, *N* = 6).

Leaf N (36.42 g kg^−1^) and P concentration (1.65 g kg^−1^) of grass and leaf P concentration (2.78 g kg^−1^) of legume PFTs under N addition (600 kg ha^−1^ NH_4_NO_3_) with Si was significantly higher than that under the same level of N addition only (leaf N and P concentration of grass PFTs is 30.25 g kg^−1^ and 1.54 g kg^−1^, and leaf P concentration of legume PFTs is 2.51 g kg^−1^ under 600 kg ha^−1^ NH_4_NO_3_ addition, respectively). Application of Si markedly increased leaf P concentration of both grass and legume PFTs (Fig. 5). There were significant interaction effects between Si and N fertilization in leaf N concentration (*F* = 4.641, *P* = 0.007) of grass PFTs (two-way ANOVA).

### Grass:legume abundance and aboveground biomass ratios

**Figure 6:**
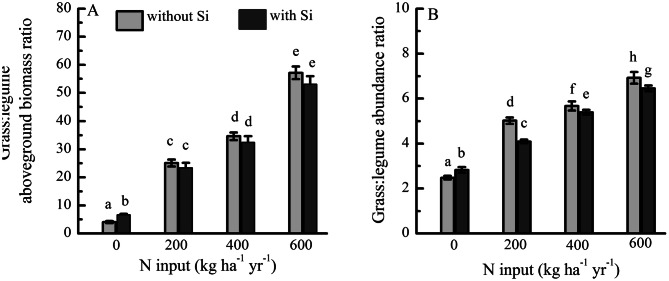
(**A**) Grass:legume ratio of aboveground biomass and (**B**) abundance under fertilization by N with Si (N + Si) and without Si (N-only) expressed as 3-year averages (2011–2013, *N* = 6).

Grass:legume aboveground biomass ratio (4.31–57.17) and abundance ratio (2.48–6.92) increased significantly with increasing rates of N addition. Silicon addition resulted in a higher ratio of the aboveground biomass and plant abundance relative to the control plots. Compared with N addition, the addition of N + Si significantly decreased the plant abundance grass:legume ratio. There was no significant difference in the aboveground biomass grass:legume ratio between the addition of N-only and the addition of N + Si (Fig. 6).

## Discussion

The addition of N did not affect the soil organic C (Table S1). This finding is consistent with several previous studies^25–27^, but contrasts with other reports^28,29^. The different findings may be attributed to (1) different amount and duration of N addition for the experimental duration. Sillen and Dieleman^27^ reported that moderate N additions promote belowground C decomposition processes, reducing the potential for increases soil C storage. Zhao et al.^29^ noted that 3-year optimization of N addition increase long-term C storage, for the experimental duration had a positive correlation with the response of soil organic carbon to N addition. (2) Different vegetation types and grassland degradation level for the response of soil C to N addition, which varied with vegetation types and the grassland degradation level^25,29^. For example, Zhao et al.^29^ noted that N addition increase soil C storage in degraded grasslands.

Silicon addition did not affect soil organic C (Table S1). This finding contrasts with recent reports^23,30,31^ that demonstrated that Si addition significantly increased soil total organic C and phytolith C sequestration. This could be because the short or long duration of the experiment for some studies found that Si plays an important role in regulating the global C balance and C turnover^32,33^. The processes of Si in regulating the C balance and C turnover involved in Si-enhanced soil organic C stability at a decadal scale include protection of soil organic C through amorphous Si^33^.

Our study also demonstrated that N fertilization did not affect soil total N. This finding was in line with a recent report^28^ but contrasted with other reports^34^. Several mechanisms could explain this discrepancy. Nitrogen addition results in N accumulation in aboveground biomass. The increasing aboveground biomass and N accumulation in aboveground biomass are often removed by livestock in vegetation types such as grasslands (alpine meadow, alpine steppe, and cultivated grassland)^28^, which is different from other vegetation types such as coniferous forest^28^. Gao et al. states that N addition promote soil total N accumulation of a cold-temperate coniferous forest^34^. Silicon addition did not affect soil total N (Table S1). This finding is inconsistent with another report that indicated that high Si addition considerably increased concentrations of N due to dissolved Si competing with other elements for binding sites at organic matter and mineral surfaces^31^. The different findings may be attributed to (1) different soil texture and (2) different Si fertilizer addition processes. Our study focused on Si-addition studies on the Qinghai–Tibetan Plateau, whereas Reithmaier et al.^31^ focused on influence of Si availability on the element concentrations in peatlands on northeastern Bavaria. Moreover, the Si was added directly to soil in our study, rather than through PVC tubes in Reithmaier et al.^31^.

Effect of N fertilization on pH decreased with N fertilization rate (Table S1), indicating that N fertilization induced soil acidification. This was probably because when an NH_4_^+^ ion is absorbed by plant roots, an H^+^ ion will be released into soil solution and cause soil acidification^26,33–36^. In contrast, NO_3_^−^ anions lead to the loss of metal cations through their leaching based on the charge balance in soil solution^37^. Silicon addition did not affect soil pH since soil pH is usually very stable in soil^38,39^.

The soil NH_4_^+^-N and NO_3_^−^-N concentrations increased with the N addition rate (Fig. 1). These results are similar to previously reported N-addition experiments performed in this region and at other sites^40,41^. However, our findings contrasted with those of some other studies. For example, Gao et al.^42^ and Song et al.^43^ found that there were no significant effects on soil NH_4_^+^-N concentrations among control and N addition plots in an alpine meadow and a Korean pine plantation. The different findings may be caused by the different environmental conditions^30,44^. The present study showed that fertilization with N + Si increased the soil NH_4_^+^-N and NO_3_^−^-N concentrations. Reithmaier et al.^31^ similarly reported that the addition of Si increased the soil available N for Si can compete with other elements for binding sites at organic matter and mineral surfaces.

The study showed that N + Si increased the amount of soil available P. This may result from the following mechanisms. First, Si can directly increase the availability of P and decrease the P retention capacity of soil by mobilizing P from unavailable phases^15,38,45^. For example, Schaller et al.^39^ found that Si is positively related to P availability and is important for mobilizing P from previously unavailable phases. Second, Si availability mobilizes P from binding sites of soil minerals^31,39^. For example, some studies found that Si strongly competes with P for binding sites in Fe minerals, with a slightly lower binding affinity of silicic acid compared with P^39^.

The increases in the aboveground biomass and abundance of grass PFTs with increasing rates of N application confirm that they are limited or co-limited by the availability of N and P in alpine meadows (Fig. 3). This has been consistently observed in other studies of alpine meadows, which found that N addition generally increased aboveground biomass of grasses and sedges on the Qinghai–Tibetan Plateau^2,6,46^. Nitrogen plus Si application increases the aboveground biomass and abundance of grass PFTs. This was probably because Si helps to increase the erectness of leaves, which, in turn, reduced self-shading and increased the net photosynthetic rate, eventually leading to an increase in the abovegroundbiomass^6,47^. Some studies have shown that Si application increases the net photosynthesis rate and water use efficiency, while the expression of some key genes related to photosynthesis is increased with Si addition even under unstressed conditions^13,30^.

Higher N fertilization rates resulted in a significant reduction in abundance and aboveground biomass of legume PFTs (Fig. 4). This was because leguminous plants can increase N availability in soils by fixing atmospheric N via symbiotic rhizobia in an available form. Therefore, leguminous plants are adapted to low-N conditions^48^. This leads to a lower abundance of leguminous plants in N-fertilized soils^1,6^, which has been consistently observed in other studies of alpine meadows^2,6,46^. The application of N + Si had a higher plant abundance and aboveground biomass than N-only. Many studies have reported that Si addition can stimulate higher rates of photo-assimilate translocation, consequently enhancing C sink strength^13,49^. Furthermore, some studies have reported that plants that accumulate Si can increase their competitiveness in herbaceous communities under nutrient-enriched soil, leading to increased abundance of leguminous plants in the community^20,48,50^.

The leaf N and P concentrations of grass and the leaf P concentration of legume PFTs were higher with Si than without Si fertilization (Fig. 5). As suggested by the growth-rate hypothesis, higher N and P concentrations in plant leaves can achieve rapid growth, and thus the growth response of the grass and legume PFTs to Si fertilization observed here could be at least partially mediated by the effect of Si on the N use efficiency and the availability of P ^13,39^. This finding agrees with a previous report examining the role of Si in growth and leaf N and P concentrations of *Phragmites australis*^16^.

Our study found that fertilization Si only increased grass:legume abundance and biomass ratios compared with the control plot (Fig. 6), which may suggest that Si can promote the growth of both grass and legumes. Our results also confirm that the addition of N + Si not only increased aboveground biomass of grass and legume PFTs, but also maintained a constant grass:legume ratio of aboveground biomass. Johnson et al.^18^ and Mali et al.^51^ observed that Si addition was beneficial not only for nodule growth but also for plant growth in terms of relative yield of root and shoot, and the increasing in root yield lead to an enhancement of root nodulation^18^. Furthermore, Si and N addition can affect grass and legume biomass production, nutrient content, and the relationship between legumes and grasses in grassland, leading to changes in plant species coexistence in the community^15–17,51^. These results suggest that application of N + Si alleviates the shift in the species composition, and promotes coexistence of grass and legume species, especially at high levels of N addition.

Grass and legume mixtures in a community can give a considerable advantage in terms of both productivity and resource utilization and widely used in grassland ecosystems^25,52^. For example, Xu et al.^53^ reported that when the grass was mixed with the legume species, the capture and absorption of N and P by the grass were greatly improved. Our study suggests that the addition of N + Si increases the amount of available P, NH_4_^+^-N and NO_3_^−^-N in the soil and the aboveground biomass of grasses and legumes relative to addition N only. This not only helps the legume to maintain its advantage in the plant community, but also improves the capture and absorption of N and P in grass and P in legume species. These results together show that Si enhances N fertilization with apparently few effects on grass:legume ratios and benefits availability of nutrients to plants, suggesting that Si is essential for the plant community in alpine meadows.

## Materials and methods

### Study site

This study was conducted in an alpine meadow at an elevation of 3,500 m above sea level on average in the Research Station of Alpine Meadow and Wetland Ecosystems of Lanzhou University, which is located on the eastern Qinghai–Tibetan Plateau (33°58′N, 101°53′E)^6^. The mean annual temperature is 1.2 °C and the mean annual precipitation is 620 mm on average from 1969 to 2005, mainly falling during the short and cool summer in this area. The plant community in this region is a typical alpine meadow with an alpine meadow soil. Soil organic matter contents, total nitrogen, total phosphorus and pH values are 70.52 g kg^−1^, 3.72 g kg^−1^, 0.98 g kg^−1^ and 6.33, respectively.

### Experimental design

In this study, the experiment was laid out inside a fence to exclude disturbance by grazing (yak and Tibetan sheep) during the plant growth season and laid out in a completely randomized block design in early May 2011. Six 16 m × 30 m blocks were selected and eight 5 m × 5 m plots were established in each block. All plots were separated by a 2 m buffer of unfertilized strips (48 plots in total, with six blocks per site). The experimental design included four different levels of N fertilization (0, 200, 400 and 600 kg ha^−1^ ammonium nitrate (NH_4_NO_3_) per year), comprising simultaneous fertilization in the plots without Si (N-only) (0 kg ha^−1^ H_4_SiO_4_ per year) or with Si (N + Si) (40 kg ha^−1^ hydrated silica (H_4_SiO_4_) per year). The fertilization took place on rainy days in early May in each year when rainfall was abundant in this region.

### Soil sampling collection and analyses

Soil samples were collected in August of 2011, 2012 and 2013 for soil nutrient analysis. The topsoil samples (0–20 cm) were collected using a bucket auger (diameter 3.8 cm, depth 20 cm) from three sites chosen at random within each plot and mixed to give a single sample. The soil samples were air-dried and any visible roots and stones removed, and then passed through a 1-mm mesh sieve. The Walkley–Black dichromate oxidation method was used to determine the soil organic C concentration. The concentrations of soil total P and available P were measured using an inductively coupled plasma spectrometer (ICP; SPECTRO ARCOS EOP, Germany). Soil total N, NH_4_^+^-N and NO_3_^−^-N concentrations were determined following the same approach as Han et al.^26^. Soil pH was measured using glass electrode in the supernatant by homogeneously mixing 5 g of soil and 25 ml of water.

### The growth of grass and legume PFTs

Plant samples were investigated from one 0.5 m × 0.5 m quadrat in every 5 m × 5 m plot when peak biomass was reached, including plant abundance and aboveground biomass. The number of all grass PFTs or of all legume PFTs was counted as grass abundance or legume abundance, respectively. All plants in each quadrat were clipped at the surface of the soil and plants from the two PFTs were selected. The dry aboveground biomass of the two PFTs in every quadrat was weighed after oven-drying at 70 °C for 48 h. The grass:legume biomass ratio was calculated as the aboveground biomass of grass PFTs/aboveground biomass of legume PFTs and the grass:legume abundance ratio was calculated as abundance of grass PFTs/abundance of legume PFTs.

### Statistical analysis

Before taking the averages of the 3 years of data (2011–2013), two-way ANOVAs (year, fertilization treatment, and interaction) were performed to determine whether there was an interaction effect of year and it was found that year had no effect on the results. Therefore, all data were presented as mean ± standard deviations of 3-year averages with six replicates in the figures. We used fertilizer amounts of N as one variable, and + or − Si as the second variable. Two-way ANOVAs (N amounts, Si, and interaction) were used to obtain an interaction effect between level of N and Si. In these cases, we then proceeded with multiple comparison tests to compare differences among means using LSD test at *P* < 0.05. Statistical analysis was conducted using SPSS 18.0 for windows (SPSS Inc., Chicago, IL, USA).

## Supplementary information

Supplementary file.
