# Supplementary material for: Silicon addition improves plant productivity and soil nutrient availability without changing the grass:legume ratio response to N fertilization
Source: Sci Rep. 2020 Jun 24;10:10295. doi: 10.1038/s41598-020-67333-7 (PMC7314743; doi:10.1038/s41598-020-67333-7)
Supplement: Supplementary file 1 — Supplementary file. [file 41598_2020_67333_MOESM1_ESM.pdf]

Silicon addition improves plant productivity and soil nutrient availability without changing the grass:legume ratio response to N fertilization

Danghui Xu[1]*, Tianpeng Gao[2,3], Xiangwen Fang[1], Haiyan Bu[1], Qiuxia Li[1], Xiaona Wang[1], Renyi Zhang[1]

[1]State Key Laboratory of Grassland Agro-ecosystems/School of Life Science, Lanzhou University, No. 222, South Tianshui Road, Lanzhou, Gansu 730000, China.

[2]College of Biological and Environmental Engineering, Xi'an University, Xi'an 710065, China.

[3]The Engineering Research Center of Mining Pollution Treatment and Ecological Restoration of G ansu

Province, Lanzhou City University, Lanzhou,730070, China

*Corresponding author email: dhxu@lzu.edu.cn

Table SI. Soil organic C, total N concentration and pH under fertilization by N with Si and without Si.

	CK	Si40	N200	N200+Si40	N400	N400+Si40	N600	N600+Si40
Soil organic C (g kg^{-1})	41.47a	41.21a	41.07a	42.24a	40.46a	41.83a	41.24a	41.29a
Total N concentration (g kg^{-1})	3.56a	3.68a	3.61a	3.72a	3.69a	3.55a	3.79a	3.82a
pH	6.33a	6.34a	6.15b	6.16b	5.98c	6.01c	5.98c	6.03c
